# Expanded population of low-density neutrophils in juvenile idiopathic arthritis

**DOI:** 10.3389/fimmu.2023.1229520

**Published:** 2023-10-17

**Authors:** Zuzana Parackova, Irena Zentsova, Marketa Bloomfield, Adam Klocperk, Rudolf Horvath, Hana Malcova, Dita Cebecauerova, Anna Sediva

**Affiliations:** ^1^ Department of Immunology, 2nd Faculty of Medicine Charles University, University Hospital in Motol, Prague, Czechia; ^2^ Department of Paediatric and Adult Rheumatology, University Hospital in Motol, Prague, Czechia

**Keywords:** juvenile idiopathic arthritis, neutrophils, low-density neutrophils, calprotectin, activation, reverse transmigration, autoimmunity

## Abstract

**Introduction:**

Juvenile idiopathic arthritis (JIA), a clinically variable disease characterized by autoimmune arthritis, affects children, and its immunopathology remains elusive. Alterations in neutrophil biology play an important role in this disease. In the present study, we aimed to explore the features of low-density neutrophils (LDNs) in patients with JIA.

**Methods:**

Gene expression of peripheral blood mononuclear cells (PBMCs) from children with distinct subtypes of JIA was analyzed by NanoString Immunology panel. Presence of LDNs was ascertained by flow cytometry and the release of neutrophil-associated products were analyzed by LUMINEX.

**Results:**

LDNs were detected in patients’ peripheral blood mononuclear cells (PBMCs) after density gradient centrifugation. Transcriptomic analysis of JIA PBMCs revealed that genes related to neutrophil degranulation were markedly upregulated. The number of LDNs and level of their degranulation products increased in patients’ PBMCs and correlated with serum calprotectin, but not with disease activity, sedimentation rate and C-reactive protein (CRP) levels. The phenotypes of LDNs varied from those of normal-density neutrophils and healthy donor LDNs. Phenotypical analysis revealed LDNs are immature and primed population with decreased suppressive capacity. A negative correlation between surface proteins CD62L, CD66b, and CD11b and the number of inflamed joints/JADAS was established.

**Conclusion:**

Our results describe LDNs as primed, degranulated, immature cells with impaired suppressive activities. This work thus contributes to the increasing body of evidence that LDNs in JIA are altered and their role in the disease immunopathogenesis and possible clinical associations should be investigated further.

## 1 Introduction

Low-density neutrophils (LDNs) are a subset of neutrophils that remain in the peripheral blood mononuclear cell (PBMC) fraction after density gradient centrifugation. Their density is similar to that of PBMCs, but different from that of normal-density neutrophils (NDNs), which segregate with other polymorphonuclear cells during density centrifugation (1). The origin and possible cellular functions of LDNs are vague and still controversial. Their presence in PBMC layer might be due to increased activation and degranulation since neutrophils are capable to change their buoyancy upon stimulation or inflammatory conditions. On the other hand, their presence might be explained by the change of cell volume which would make LDNs not activated/degranulated neutrophils, but rather primed neutrophils (2). The LDN phenotypical characterizations also continues to be challenging since there is no specific marker that allows to distinguish LDNs from granulocytic myeloid derived suppressive cells (G-MDSC) (3). They are even considered to be the same population by some, and a functional assay to confirm their immunosuppressive properties appears to be a good discriminative option (4).

Low-density neutrophils were first described in systemic lupus erythematosus (SLE) (5), and their count correlates with the severity of vasculitis. Since then, LDNs have been identified in cancer (6, 7), other autoimmune diseases (8–10), infections (8–11), and inflammation (12, 13) where they are speculated to contribute to disease pathology. LDNs have varying functions among inflammatory disorders, indicating the disease-specific LDN diversity. In SLE, LDNs can damage endothelial cells and produce pro-inflammatory cytokines (1), whereas in rheumatoid arthritis (RA), LDNs are not as functionally efficient as NDNs (9). Nevertheless, LDNs are considered highly pro-inflammatory because of their ability to spontaneously produce neutrophil extracellular traps (NETs), reactive oxidative species (ROS), cytokines, and display endothelial toxicity. Their presence is often associated with the severity of several immune-mediated diseases (14). Also, it is possible that LDN functions vary among different diseases. In SLE, LDNs can activate T cells (10), on the other hand, in cancer, LDNs are capable inhibiting T cell responses and promote tumor growth and metastization (15). Because of their immunosuppressive properties, they represent a potential target for immunotherapies focused on disinhibition of the effector cytotoxic T lymphocytes (16).

Juvenile idiopathic arthritis (JIA) is commonly considered to be a multifactorial autoimmune disease driven by a combination of environmental triggers and genetic susceptibility. It is characterized by arthritis that lasts for more than 6 weeks and occurs before 16 years of age. The most common form of JIA is oligoarticular arthritis affecting up to four joints, followed by the polyarticular form (>5 joints), enthesopathy-related arthritis (ERA), systemic JIA (sJIA), and rheumatoid factor (RF)-positive polyarthritis. The JIA subtypes differ not only by clinical symptoms but also by the immunopathogenesis. Most children first manifest the symptoms at around 3 or between 6 and 12 years of age (17–20).

The pathophysiology of JIA is still not completely understood. The key role of neutrophils in the early steps of the pathological immune response has been elucidated (21–25). Neutrophils are the most prevalent cells in the synovial fluid of inflamed joints and exhibit signs of hyperactivation and an impaired capacity to suppress T cell proliferation. In addition, they exhibit defective oxidative bursts and phagocytic activity (21, 22, 26). Notably, using public microarray datasets of PBMCs collected from patients with JIA, a neutrophil activation signature has been identified and the possible involvement of LDNs in JIA pathology has been reported (25).

Therefore, in the present study, we determined the number of LDNs in patients with JIA and focused on their deeper characterization, particularly their phenotypic and functional differences, and their correlation with clinical assessments of patients.

## 2 Methods

### 2.1 Patients

Patient blood samples were obtained from the Department of Paediatric and Adult Rheumatology, University Hospital in Motol, where they have been followed and fulfilled the International League of Association for Rheumatology’s (ILAR) criteria. The study involved 15 pediatric patients with JIA (juvenile arthritis disease activity score-71: JADAS71 ≥ 1; 20% male individuals; median age 12.88 ± 4.10 years) and 17 healthy donors (40% male individuals; mean age 15.50 ± 9.48 years). The JADAS consists of four elements: 1) the active joint count, 2) physician global evaluation, 3) parent/patient visual analog scale of well-being (VAS), and 4) erythrocyte sedimentation rate (ESR) (27). The patients were recruited to this study based on the following criteria: patients with oligoarticular/oligoarticular-extended, polyarticular and ERA JIA subtypes with the active state of disease (JADAS71 ≥ 1) who were approved for the anti-TNF-α treatment. No patients with systemic JIA (sJIA), psoriatic arthritis and undifferentiated arthritis were included in this study. To assess LDNs in different JIA subtypes with likely variable immunopathogenesis, we compared the data between the three JIA subgroups to ensure that we described general mechanisms shared between the pediatric patients with chronic autoimmune inflammatory arthritis. The healthy donors had no previous history of autoimmune diseases. The patient demographic attributes are summarized in **Table 1**.

**Table 1:** Patient cohort characteristics.

COHORT	ALL	oligoarticular	polyarticular	Enthesitis-related arthritis
Demography				
Patients (n)	15	5	5	5
Gender (males)	3	1	1	1
Age (median, +/-SD, years)	12.88 ± 4.10	10.46 ± 4.21	10.66 ± 5.25	14.52 ± 1.94
Disease characteristics				
Antinuclear antibodies positivity	7	2	4	1
Human leukocyte antigen B27 positivity	5	0	2	3
Rheumatoid factor positivity	0	0	0	0
Anti-citrullinated protein antibody positivity	0	0	0	0
Disease activity				
active joint count (median +/- SD)	4.00 ± 3.18	4.2 ± 1.92	8.5 ± 3.32	3.4 ± 2.2
active uveitis	2	2	0	0
Erythrocyte sedimentation rate (mean +/- SD, mm/hour)	13.50 ± 8.12	10.43 ± 4.47	12.00 ± 12.17	17 ± 9.13
C-reactive protein (mean +/- SD, mG/L)	4.85 ± 8.94	11.92 ± 13	4.93 ± 4.00	2.76 ± 2.64
Juvenile arthritis disease activity score-71	8.43 ± 7.26	13.00 ± 2.55	16.75 ± 5.74	7.8 ± 3.37
Treatment				
Glucocorticoid	4	2	1	1
Conventional synthetic disease-modifying antirheumatic drug	15	5	5	5
Anti-tumor necrosis factor-alpha	0	0	0	0
Neutrophil counts				
Relative numbers (mean +/- SD; HD range 0.45-0.7)	0.55 ± 0.098	0.57 ± 0.12	0.55 ± 0.05	0.54 ± 0.08
Absolute numbers (mean +/- SD; x10*9/l; HD range 2.00-7.00)	3.95 ± 1.87	4.29 ± 2.37	3.58 ± 1.50	4.06 ± 0.94

**Baseline characteristics of the study cohort:** 15 enrolled patients with juvenile idiopathic arthritis (JIA) with active disease (JADAS71 ≥ 1). SD, standard deviation.

Written informed consent was obtained from all patients or their parents/guardians in accordance with the tenets of the Declaration of Helsinki, and the study was approved by the Ethics Committee of University Hospital in Motol.

### 2.2 Cell isolation

Peripheral blood was collected in EDTA-coated tubes, and then PBMCs were isolated using Ficoll–Paque (GE Healthcare BioSciences, Uppsala, Sweden) and washed three times in phosphate buffered saline (PBS). The isolated PBMCs were cultured in a RPMI 1640 medium (Invitrogen, Carlsbad, CA, USA) supplemented with 10% FBS, 1% penicillin, and 1% GlutaMAX (Thermo Fisher Scientific, Waltham, MA, USA).

### 2.3 RNA extraction and quality assessment

The total RNA was extracted from PBMC pellets stored at –80°C using the RNeasy MiniKit (Qiagen, Hilden, Germany) isolation kit following the manufacturer’s instructions. The RNA was quantified using NanoDrop, and the quality of RNA was determined using a TapeStation 4200 (Agilent, St. Clara, CA USA).

### 2.4 Gene expression analysis using NanoString profiling

Approximately 100 ng of total RNA was used to measure the expression of 730 immunity-related genes, and 40 housekeeping genes using the nCounter platform (NanoString Technologies) and the Immunology panel. Data was analyzed by ROSALIND^®^. All samples satisfying quality assurance metrics checks were log- transformed (base 2) to help with distributional assumptions and normalized using housekeeping genes. Differentially expressed genes (DEGs) were determined as genes with a p value ≤ 0.05 and a false discovery rate (FDR) ≤ 5%. P-value adjustment was performed using the Benjamini-Hochberg method by estimating FDR. Hypergeometric distribution was used to analyze the enrichment of pathways. REACTOME database source was referenced for enrichment analysis. The gene enrichment was calculated relative to a set of healthy donors’ genes.

### 2.5 LDN and NDN determination

Peripheral blood was incubated with a mixture of antibodies containing anti-lineage specific markers (CD3 clone MEM-57, CD19 clone LT19, CD20 clone LT20, CD56 clone MEM-188, CCR3 clone 5E8)-FITC, CD10-PEDY594 (clone MEM-78) all from Exbio (Prague, Czech Republic); CD66b-PC7 (clone G10F5), CD62L-BV650 (clone DREG-56), CD14-APC (clone HDC14), CD11b-BV510 (clone ICRF44), CD33-BV421 (clone P67.6), CD16-A700 (clone 3G8), and PDL1-PE (clone 29E.2A3) from Biolegend (San Diego, CA, USA); and HLA-DR-PerCP (clone 243) from BD Biosciences (San Jose, CA, USA) for 20 min and then hypotonically lysed. Samples were acquired on BD Fortessa and analyzed using FlowJo software.

### 2.6 Neutrophil degranulation determination

Isolated 10^6^/ml PBMCs were left untreated in 37°C overnight. The cell- free supernatant was collected and stored at -20°C until further analysis. For detection of degranulation products (lipocalin, matrix metalloproteinase 8, lactoferrin, myeloperoxidase and proteinase 3) multianalyte profiling was performed using the LUMINEX xMAP technology (R&D Systems).

### 2.7 Serum calprotectin

The clotted whole blood was centrifugated for 10 minutes at 3000 rpm. The serum was then collected, aliquoted and stored at -80°C until further analysis. Human calprotectin (S100A8/A9) was determined by ELISA (Abcam, Cambridge, UK).

### 2.8 Statistical analysis

The obtained data from at least four independent experiments are presented as median values. Not all patients were involved in all experiments because of the limited blood amount. The results were analyzed using a non-parametric one-way analysis of variance with Dunn’s *post-hoc* test for multiple comparisons where applicable. A two-tailed paired Wilcoxon or unpaired Mann–Whitney *t*-test was also used for data analysis using GraphPad Prism 8. Results with p < 0.05 (*), p < 0.01 (**), p < 0.001 (***), and p < 0.0001 (****) were considered statistically significant.

## 3 Results

### 3.1 Neutrophil degranulation was enriched in the PBMCs from patients with JIA

15 pediatric patients with JIA and 17 healthy donors were involved in this study. All JIA patients have active state of the disease, i.e., JADAS71 ≥ 1, and comprised patients with oligoarticular/oligoarticular-extended, polyarticular, and ERA JIA subtypes.

**Figure 1:**
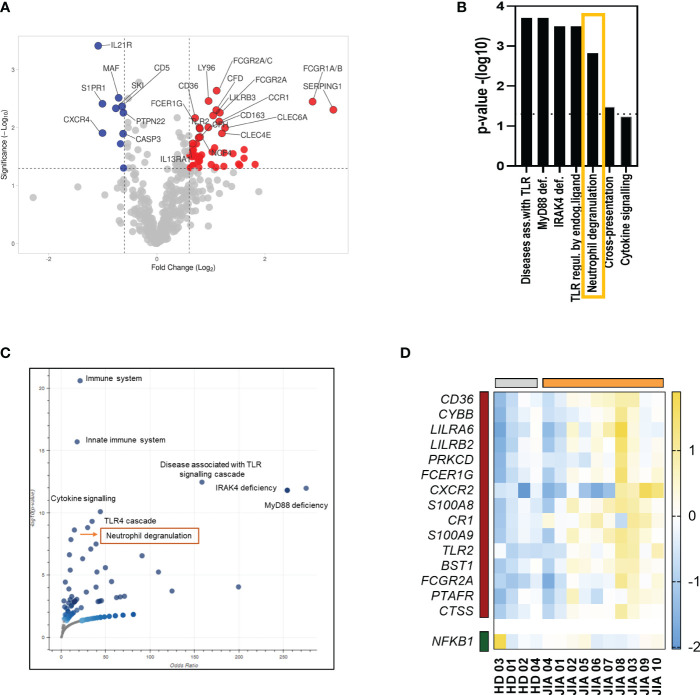
Transcriptomic analysis of PBMCs from patients with JIA. **(A)** volcano plot showing upregulated and downregulated DEGs in PBMCs from patients with JIA compared with that in PBMCs from HDs. **(B)** Enriched pathways in PBMCs in patients with JIA. **(C)** A volcano plot of enriched terms in the Bioplanet 2019 gene set library. Each point represents a single term in the library, plotted by the corresponding odds ratio (X-position) and -log10 (p-value) (Y-position) from the enriched DEGs. **(D)** Heatmap of DEGs involved in neutrophil degranulation. JIA, juvenile idiopathic arthritis; PBMC, peripheral blood mononuclear cell; DEG, differentially expressed gene; HD, healthy donor.

The transcriptional analysis of PBMCs from patients with JIA and HDs revealed 57 differentially expressed genes (DEGs) (**Figure 1A**). These DEGs play roles in various biological processes but are mostly involved in cytokine and TLR signaling. One of the most markedly enriched pathways in PBMCs from patients with JIA was neutrophil degranulation (**Figures 1B, C**). A total of 1 downregulated and 15 upregulated genes were found to be involved in neutrophil degranulation (**Figure 1D**).

### 3.2 Low-density neutrophils were expanded in PBMCs from patients with JIA

**Figure 2:**
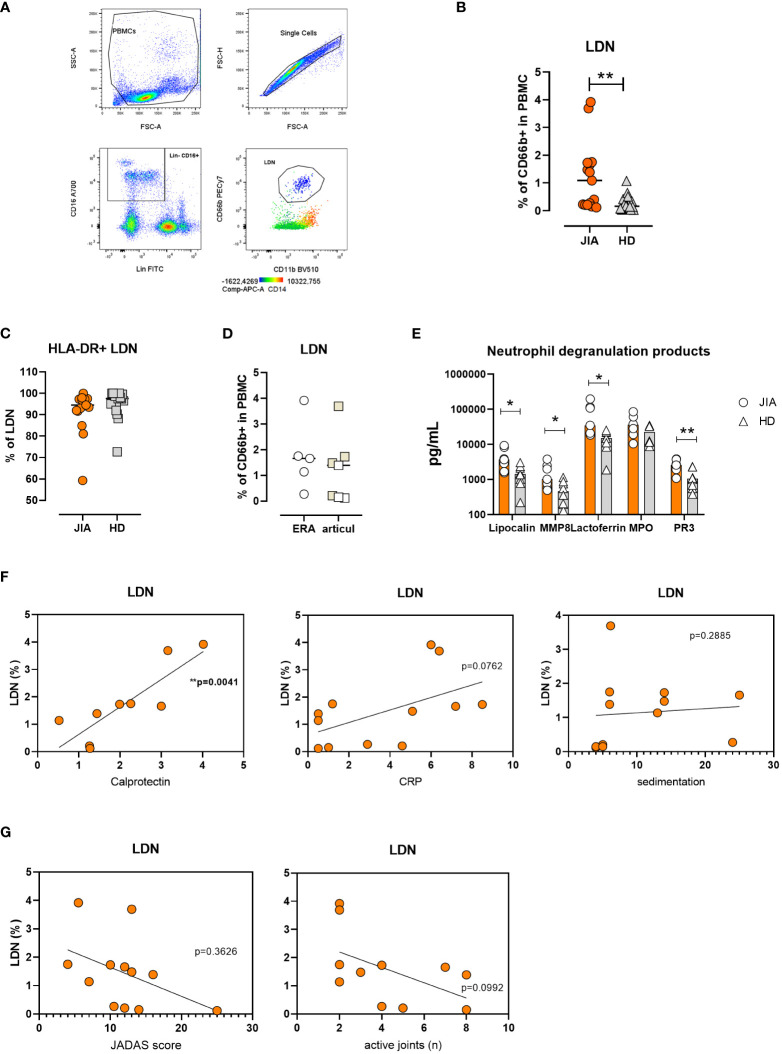
Low-density neutrophils in JIA. **(A)** Gating strategy for LDN identification. **(B)** Quantification of LDNs in patients with JIA (n = 15) and HDs (n = 17) **(C)**. % of HLA-DR+ LDNs in patients with JIA (n = 15) and HDs (n = 17) **(D)**. LDN percentage in JIA subtypes: oli-polyarticular and ERA groups. **(E)** Neutrophil degranulation products in PBMC cultures. **(F)** Correlation between LDN percentage in patients with JIA and serum calprotectin, CRP level and sedimentation rate. **(G)** Correlation between LDN percentage in patients with JIA, JADAS and the number of inflated joints. JIA, juvenile idiopathic arthritis; LDN, low-density neutrophil; HD, healthy donor; ERA, enthesopathy-related arthritis; PBMC, peripheral blood mononuclear cell; MMP8, matrix metalloproteinase 8; PR3, proteinase 3; CRP, C-reactive peptide; JADAS, juvenile arthritis disease activity score. Values are standardized and expressed as median values. Statistical analyses were performed using paired *t*-tests and linear regression. Values with p < 0.05 (*) and p < 0.01 (**) were considered significant.

The transcription analysis indicated the presence of neutrophils in the PBMC fraction, which prompted us to investigate the presence of LDNs. The cell population was defined as Lin-CD16+CD11b+CD66b+ CD14- cells (**Figure 2A**). We found increased percentage of LDNs in patients with JIA compared to healthy donors (HDs) (median: JIA 1.09% ± 1.23% vs. HD 0.17% ± 0.28% of total PBMCs) (**Figure 2B**). LDNs expressed HLA-DR with no differences between patients with JIA and HDs (**Figure 2C**). We compared the LDN percentages between patients with the articular form of JIA and those with ERA and observed no differences between the groups (**Figure 2D**). Similarly, no correlation was found between the LDN percentages and patients’ age (**Supplementary Figure 1A**).

To further confirm LDN presence in PBMCs from patients with JIA, we cultured PBMCs in complete medium for 24 h and detected neutrophil degranulation products using LUMINEX. Our analysis revealed increased levels of lipocalin, matrix metalloproteinase 8 (MMP8), lactoferrin, and proteinase 3 (PR3) in patients’ PBMC cultures (**Figure 2E**).

### 3.3 LDN percentage correlated with serum calprotectin levels but not with clinical severity

We were intrigued whether elevated LDN percentage in JIA correlates with other routinely used markers of inflammation. Therefore, we compared the number of LDNs with serum calprotectin (S100A8/A9), C-reactive protein (CRP) level and sedimentation rate. The linear regression analysis showed a positive correlation of LDNs with calprotectin and no correlation with CRP and sedimentation rate in the patients (**Figure 2F**).

Similarly, no significant relationship was observed between clinical severity (number of active joints and JADAS score) and LDN percentage (**Figure 2G**).

### 3.4 LDNs markedly differed from NDNs

**Figure 3:**
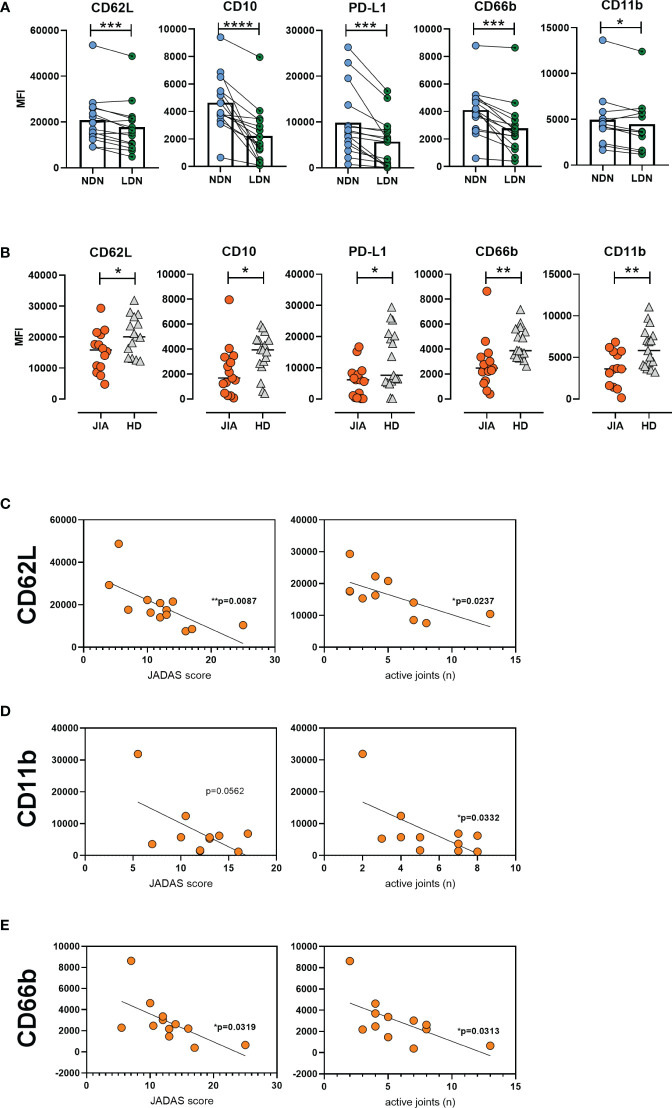
Low-density neutrophil characteristics in JIA. **(A)** Surface marker expression on LDNs and NDNs in matched samples (n = 15). **(B)** LDN phenotype in patients with JIA (n = 15) and HDs (n = 17). Correlation between **(C)** CD62L **(D)** CD11b, and **(E)** CD66b expression and JADAS and number of active joints. JIA, juvenile idiopathic arthritis; LDN, low-density neutrophils; NDN, normal-density neutrophil; HD, healthy donor; JADAS, juvenile arthritis disease activity score. Values are standardized and expressed as median values. Statistical analyses were performed using paired *t*-tests and linear regression. Values with p < 0.05 (*), p < 0.01 (**), p < 0.001 (***), and p < 0.0001 (****) were considered significant.

Next, we aimed to characterize LDNs in detail; thus, we compared their features with those of NDNs in matched patient samples. The expression of CD62L was low on LDN surface, suggesting a more active and primed state of LDNs. Low CD10 level implied an immature state and decreased PD-L1 expression, suggested that LDNs were less suppressive than NDNs (**Figure 3A**). In addition, the expression of the degranulation and adhesive marker CD66b on LDN surface decreased when compared to that on the NDN surface; and CD11b expression decreased as well but not as significantly as CD66b (**Figure 3A**). We observed similar differences between NDNs and LDNs in matched healthy samples. We compared CD11b and CD66b expression between HLA-B27 negative JIA patients (articular form only) and those with ERA and observed no differences between the groups (**Supplementary Figure 1B**).

### 3.5 Phenotype of LDNs from patients with JIA varied from that of LDNs from HDs

We compared the LDN phenotype between patients with JIA and HDs. The expression of CD62L on JIA LDNs was low, indicating their higher activated status. Similarly, decreased surface CD10 level suggested a more immature state and decreased PD-L1 expression indicated the less suppressive profile of LDNs from patients with JIA (**Figure 3B**). Moreover, we detected significantly decreased levels of CD66b and CD11b on the surface of LDNs from patients with JIA (**Figure 3B**). We observed a negative correlation between CD62L expression and JADAS and the number of active joints in patients with JIA (**Figure 3C**). We observed a similar relationship between CD11b and CD66b surface expression and JADAS and the number of active joints in patients with JIA (**Figures 3D, E**). No relationship was observed between CD11b and CD66b expression and inflammatory parameters (CRP level and sedimentation rate) (**Supplementary Figure 1C**).

## 4 Discussion

In this study, we identified and characterized LDNs in the PBMCs of patients with JIA using transcriptomic analysis and flow cytometry. LDNs from patients with JIA were phenotypically distinct from NDNs. In addition, LDNs from patients with JIA were increased and showed altered expression of multiple surface molecules compared to LDNs from HDs. Notably, the expression of CD62L, CD66b, and CD11b on the surface of LDNs from patients with JIA was decreased and negatively correlated with the number of inflamed joints and JADAS.

The analysis of publicly available datasets of RNA isolated from PBMCs of patients with JIA indicated the presence of neutrophils in the PBMC fraction (25). In addition, we suspected neutrophil infiltration into the PBMC fraction as the transcription analysis identified neutrophil degranulation as one of the most significantly upregulated processes in PBMCs from patients with JIA. Also, the elevated LDN percentage in JIA has already been reported (25), and although we employed different gating strategies, we arrived at the same conclusion. Neutrophils migrate to the site of inflammation or activate during inflammation, they release biologically active enzymes (28). The levels of these enzymes such as MPO, NE, and MMP8 reportedly increased in the sera of patients with JIA (23). We noted that these enzymes were overproduced in the JIA patients’ PBMC, presumably by LDNs. This is important, because the presence of elevated neutrophil-associated products may contribute to the inflammatory milieu and tissue damage. Moreover, there is evidence of elevated protease levels in adult rheumatoid arthritis (RA) synovial joints (29, 30), therefore a similar pattern may occur in the inflamed joints of patients with JIA.

The origin of LDNs remains unclear. They might represent a completely distinct neutrophil population, as observed in SLE, where they are considered an aberrantly developed lineage of neutrophils resulting from genomic damage (8, 31). In contrast, LDNs may represent neutrophils with the lowest density within a spectrum of buoyant densities, found even in healthy individuals. Inflammatory conditions can lead to a reduction of density of all neutrophils (32, 33). Similarly, neutrophils can shift their density spectrum upon activation (8, 29), and only in cells on the lower end of the density range will occur in the PBMC fraction (34).

The presence of neutrophils in the PBMC fraction has been described in several pathological circumstances; yet, their characterization is inconclusive despite having employed various morphological, functional, and phenotypical markers (35). Both pro- and anti-inflammatory properties and immature and mature morphologies of LDNs have been identified (35). The results of the transcriptomic analysis of LDNs from patients with RA suggested that they resemble immature neutrophils (1, 9). In our hands, cells from JIA patients display distinct immature features. Of note, the immaturity of LDNs is supported by the fact that band immature neutrophils have a lower density (36, 37). The expansion of immature neutrophils has been previously described in patients with JIA (23). Emergency granulopoiesis often occurs during acute and chronic inflammation, leading to the appearance of the immature forms of neutrophils in the circulation. As neutrophil progenitors and early stage neutrophils have high buoyancy, they can occur in the PBMC fraction (38, 39).

The density of healthy mature neutrophils decreases upon *in vitro* stimulation. Under inflammatory conditions, mature neutrophils can be found in the PBMC fraction, indicating that inflammatory LDNs are mature and activated neutrophils. They often show signs of activation such as increased expression of CD11b and CD66b compared with that in NDNs, as described in patients with advanced adenocarcinoma (40). We observed a decrease in CD62L expression in our JIA cohort, implying an activated state of LDNs in patients with JIA. However, it cannot be excluded that *in vivo* LDNs are primed and that they subsequently become more susceptible to other stimuli. JIA LDN percentage positively correlated with serum calprotectin levels, a potential marker for diagnosis, prediction of disease relapse, response to treatment and risk of flares in JIA and other autoimmune diseases, such as RA, SLE and inflammatory bowel disease (41–43). Calprotectin is released mostly by phagocyting cells such as neutrophils at the site of inflammation, where it can modulate inflammatory response (44). It is uncertain whether LDNs contribute to the increased calprotectin levels or whether calprotectin activates neutrophils, and they consequently decrease their density and occur in the PBMC fraction. The latter would suggest that systemic inflammation may be the cause of LDN expansion in JIA, however, in our experiments LDN percentage in patients with JIA did not correlate with neither sedimentation rate nor CRP. Degranulation may be another cause of density change. Measurements of granule markers on LDNs, such as CD63, CD11b, and CD66b, supported this concept (40, 45). However, several arguments have been raised against this notion, including the fact that the granular content of LDNs does not demonstrate signs of extensive degranulation (8). In addition, the majority of *in vitro* activated neutrophils revert to their original density after a couple of hours (46). In this study, LDNs from patients with JIA did not express increased levels of surface granule markers, implying that they had normal granule content.

Neutrophil migration to the sites of infection or injury is a crucial step in innate immunity, and neutrophil retention can lead to chronic inflammation and tissue damage. Activated neutrophils at the sites of inflammation do not necessarily undergo apoptosis and clearance by macrophages and in some circumstances, they may undergo reverse migration and re-enter circulation (reverse transendothelial migration) (47–49). Neutrophils, including their activated and degranulated fractions, arrive at the affected tissues where they perform multiple functions. Neutrophils are the most abundant cells found in synovial fluid of JIA patients and we hypothesized that their LDNs might represent a transmigrated neutrophil subset from the sites of inflammation into the joints (11, 35, 50). Transmigrated neutrophils account for 1–2% of circulating neutrophils in patients with RA, where they produce high amounts of ROS (51). They are hallmarked by a distinctive phenotype of CD54-hi CXCR1-low (51) and decreased expression of CD62L and CD11b (51). The latter two markers were found significantly decreased in the LDNs of patients with JIA in our study, which implies their possible transmigratory origin. Moreover, the expression of CD62L, CD11b, and CD66b negatively correlated with the number of active joints, suggesting that transmigration might be the reason for their decreased expression on the LDN surface in patients with JIA, and that their activated phenotype might be acquired in other tissues. Of note, neutrophils in the tissue may have a phenotype similar to *in vitro* activated neutrophils (51, 52).

The main limitations in the presented data interpretation are the size and heterogeneity of the studied JIA patient cohort, required for statistical strength to allow robust clinical and cellular phenotype correlations. Four patients were treated with glucocorticoids, which may have affected the neutrophil biology. The data regarding transmigration is indirect, and a phenotypic analysis of transmigrated neutrophils and neutrophils infiltrating the synovial fluid should be performed to confirm the hypothesis of reverse migration of neutrophils. Moreover, functional and morphological studies of LDNs in JIA are lacking. Methodologically, the quantification of degranulation products from Luminex analysis, as well as the analysis of RNA expression performed in PBMC, might be affected by other cells originating from PBMCs.

Several reports have indicated the critical role of neutrophils in JIA immunopathogenesis. Synovial neutrophils exhibit an activated state and impaired functions such as ROS production, phagocytosis, and T cell suppressive features (21, 22). Furthermore, neutrophils in the peripheral blood of patients with JIA demonstrated substantial variations in their subset distribution, phenotype, and a tendency to form aggregates with platelets (PNAs), which affect their activation and pro-inflammatory features (23, 53, 54).

This study contributes to the mounting evidence that LDNs in JIA are altered in their counts, phenotype and functions and may therefore be important players in the disease development or progression. Specifics of their origin, possible contribution to site-specific inflammation, functional characteristics and clinical applications should be addressed by future studies. For instance, the percentage of LDN might be helpful as a biomarker to know subclinical joint inflammation. Larger-sized cohorts should assess whether LDN/LDN features are superior as a marker over other biomarkers, such as calprotectin.

## Data availability statement

The raw data supporting the conclusions of this article will be made available by the authors, without undue reservation. The data presented in the study are deposited in the GEO repository, accession number GSE235572.

## Ethics statement

The studies involving humans were approved by the Ethics Committee of University Hospital in Motol. The studies were conducted in accordance with the local legislation and institutional requirements. Written informed consent for participation in this study was provided by the participants’ legal guardians/next of kin.

## Author contributions

ZP designed the study, performed the experiments, statistical and graphical analyses, and wrote the manuscript. IZ performed PBMC culture and nanoString experiments. MB and AK provided healthy donor information, biological material and reviewed the manuscript. RH, HM, and DC provided patient information, biological material and reviewed the manuscript. AS obtained the funding and reviewed the manuscript. All authors contributed to the article and approved the submitted version.
