# Supplementary material for: Expanded population of low-density neutrophils in juvenile idiopathic arthritis
Source: Front Immunol. 2023 Oct 17;14:1229520. doi: 10.3389/fimmu.2023.1229520 (PMC10616245; doi:10.3389/fimmu.2023.1229520)
Supplement: Supplementary file 1 [file DataSheet_1.pdf]

Expanded population of low-density neutrophils in juvenile idiopathic arthritis

Zuzana Parackova[1]*, Irena Zentsova[1], Marketa Bloomfield[1], Adam Klocperk[1], Rudolf Horvath[2], Hana Malcova[2], Dita Cebecauerova[2], Anna Sediva[1]

[1]Department of Immunology, 2nd Faculty of Medicine Charles University, University Hospital in Motol, V Uvalu 84, Prague, Czech Republic

[2]Department of Paediatric and Adult Rheumatology, University Hospital in Motol, V Uvalu 84, Prague, Czech Republic

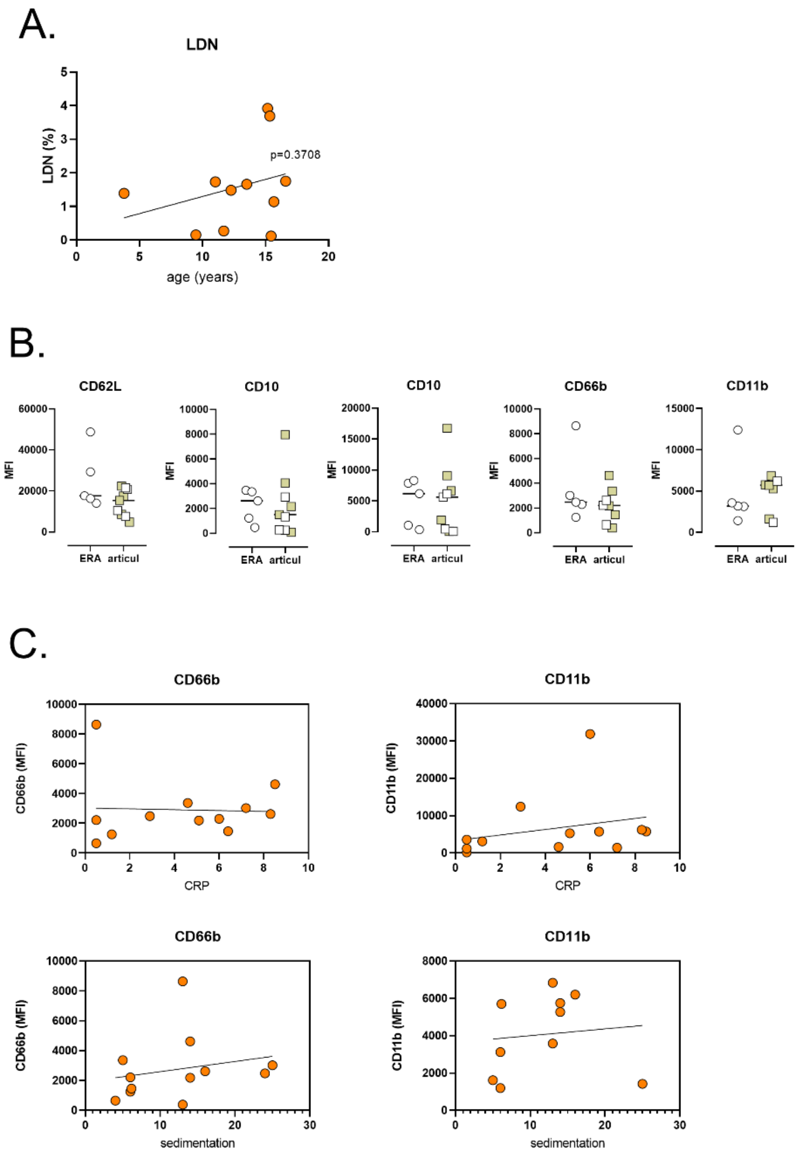

Supplementary Figure: A. Correlation between LDN count and patient age. **B.** CD11b and CD66b expression on LDNs in patients with ERA or articular only form C. Correlation between CD66b and CD11b with CRP and sedimentation rate in JIA patients

LDN, low-density neutrophils; JIA- juvenile idiopathic arthritis; ERA, enthesis-related arthritis; CRP, C-reactive protein. Values are standardized and expressed as median values. Statistical analyses were performed using paired *t*-tests and linear regression. Values with $p < 0.05$ (*), $p < 0.01$ (**), $p < 0.001$ (***), and $p < 0.0001$ (****) were considered significant.
